# Initial level and rate of change in grip strength predict all-cause mortality in very old adults

**DOI:** 10.1093/ageing/afx087

**Published:** 2017-05-25

**Authors:** Antoneta Granic, Karen Davies, Carol Jagger, Richard M. Dodds, Thomas B L Kirkwood, Avan A Sayer

**Affiliations:** 1 AGE Research Group, Institute of Neuroscience, Newcastle University, Newcastle upon Tyne, UK; 2 NIHR Newcastle Biomedical Research Centre, Newcastle University, and Newcastle upon Tyne Hospitals NHS Foundation Trust, Newcastle upon Tyne, UK; 3 Newcastle University Institute for Ageing, Newcastle upon Tyne, UK; 4 Institute of Health and Society, Newcastle University, Newcastle University, Newcastle upon Tyne, UK; 5 Academic Geriatric Medicine, Faculty of Medicine, University of Southampton, Southampton, UK; 6 Institute for Cell and Molecular Biosciences, Newcastle University, Newcastle upon Tyne, UK; 7 MRC Lifecourse Epidemiology Unit, University of Southampton, Southampton, UK; 8 NIHR Collaboration for Leadership in Applied Health Research and Care, Wessex, University of Southampton, Southampton, UK

**Keywords:** ageing, cohort study, mortality, risk factor, grip strength, grip strength decline, older people

## Abstract

**Objective:**

to investigate the associations between initial level and rate of change in grip strength (GS) and all-cause mortality in very old adults (≥85 years) over a 9.6-year follow-up.

**Methods:**

prospective mortality data from 845 participants in the Newcastle 85+ Study were analysed for survival in relation to GS (kg, baseline and 5-year mean change) using Cox proportional hazards models.

**Results:**

during the follow-up, 636 (75.3%) participants died. Higher baseline GS was associated with a decreased risk of mortality in all participants [hazard ratio (HR) = 0.95, 95% confidence interval (CI): 0.93–0.98, *P* < 0.001], men (HR = 0.97, 95% CI: 0.95–0.99, *P* = 0.009) and women (HR = 0.96, 95% CI: 0.94–0.99, *P* = 0.007) after adjustment for health, lifestyle and anthropometric factors. Overall GS slope had a downward trajectory and was determined in 602 participants: 451 experienced constant decline (negative slope) and 151 had increasing GS (positive slope) over time. Men and women with a negative slope had a 16 and 33% increased risk of mortality, respectively, with every kg/year decline in GS (*P* ≤ 0.005), and participants with a positive slope had a 31% decreased risk of mortality (*P* = 0.03) irrespective of baseline GS and key covariates.

**Conclusion:**

higher baseline GS and 5-year increase in GS were protective of mortality, whilst GS decline was associated with an increased risk of mortality in the very old over 9.6 years, especially in women. These results add to the biological and clinical importance of GS as a powerful predictor of long-term survival in late life.

## Introduction

Normative population-based studies have established a lifespan trajectory of grip strength (GS), peaking in early adulthood, maintained in middle life and steadily declining in late life [1, 2]. Weak GS—values below the age and sex-specific norms have been linked to adverse health outcomes including mobility decline [3], disability [4] and mortality in middle and late adulthood [5–11]. For example, findings from a global population-based study involving urban and rural areas from 17 countries, concluded that every 5 kg lower GS at baseline was associated with a 16% increased risk of all-cause and 7–17% greater risk of cause-specific mortality in 140,000 participants aged 35–70 over a 4-year follow-up [7].

GS as a measure of upper body strength correlates well with overall muscle strength [12], and is lost much faster after the age of 80 [1], and more in men than women [13], thus reflecting the increasing vulnerability to poor health and functioning in later life. Although epidemiological evidence supports GS as a simple, inexpensive screening tool for identifying increased risk of mortality in older adults, its predictive value is less exploited in the very old (aged 85 and over), despite the highest prevalence of muscle weakness in this age group. For example, about 56% older adults aged ≥80 in the BELFRAIL study had weak GS [14], and over 74% of the very old in the Newcastle 85+ Study had either low GS or slow gait speed [15]. In the present study we investigated the discriminatory ability of initial level and rate of change in GS over time for long-term mortality in the very old. We postulated that for both men and women the mean (absolute) decline in GS per year (slope) may be more predictive of mortality than baseline muscle strength (GS), regardless of its initial level.

Findings from the studies using the rate of change in muscle strength and physical performance as predictors of mortality have been mixed [13, 16–23]. For example, in the Baltimore Longitudinal Study (BLSA) the rate of change in GS in men aged < 60 was more important for long-term mortality than initial level, whilst for men aged ≥60 higher baseline GS but not slope predicted longer survival over a 40-year period [18].

To determine the importance of GS for survival in later life, we investigated whether initial level or change in GS were predictive of long-term mortality in the very old (aged ≥85) and whether associations differed by sex.

## Methods

### Study cohort

Participants belonged to the Newcastle 85+ Study, a prospective cohort study of the very old living in North East England (for details see Appendix 1, available in *Age and Ageing* online). The study examined health and functioning of a cohort born in 1921, and collected a range of biological, social, and psychological measures at baseline in 2006/07 (wave 1), 1.5 (wave 2), 3 (wave 3) and 5-year follow-up (wave 4) as described [24, 25]. At baseline 845 participants had data from multidimensional health assessments and general practice medical records review (GPrr). Of those, 813 (96.2%) had baseline GS measurement, followed by 605 (71.6%), 452 (53.5%) and 294 (34.6%) at waves 2–4, respectively. The study was approved by the Newcastle & North Tyneside Local Research Ethics Committee One.

### Mortality data

Date of death was obtained through the UK Health and Social Care Information Centre and confirmed by family members for a few participants. Survival time (years) was calculated from the date of baseline GS assessment to the date of death or censoring (6 January 2016). The mean (SD) follow-up time for the 845 participants was 5.35 (3.03) years with a maximum of 9.56 years.

### GS measurements

Isotonic GS as a measure of upper body and overall muscle strength [12] was assessed using a standardised protocol at baseline and each follow-up using a hand-held dynamometer (Takei hand-held Model A5401; Takei Scientific Instruments Co., Ltd., Niigata City, Japan) as described [26]. Two measurements for each hand were recorded, and the mean (*M*, SD) of four measurements (in kg) calculated. Baseline GS was used as continuous variable and categorised in sex-specific quartiles (Q1–Q4; shown in Supplementary data, Table S1, available in *Age and Ageing* online). Over 5 years 606 (71.7%) participants had ≥2 GS assessments from any wave.

### GS slopes over 5 years (absolute change)

Rate of change in GS per year (slope) was calculated for all participants with at least two consecutive GS assessments. Of those with ‘overall’ slope determined (*n* = 602), 451 (74.9%) experienced negative slopes and 151 (25.1%) had positive slopes or no change in GS. Further details are described in Supplementary data, Appendix 1, available in *Age and Ageing* online. The slopes were treated as continuous, and also categorised in sex-specific tertiles (T1–T3; shown in Supplementary data, Table S2, available in *Age and Ageing* online).

### Confounders

Confounders common to literature investigating mortality risk and GS were considered for inclusion in the models [6, 7, 10, 16, 18]. For description of confounders see Supplementary data, Appendix 1, available in *Age and Ageing* online. We included: (i) heath-related factors (self-rated health, cognitive impairment, depressive symptoms) [25] and multi-morbidity; (ii) lifestyle factors (current alcohol intake and physical activity) [27] and (iii) anthropometry [height (in cm) and fat mass (in kg)] [28]. Categorical and continuous confounders (0.1–7% missing data) were imputed with the referent category or sex-specific mean, respectively, see Supplementary data, Appendix 1, available in *Age and Ageing* online.

## Statistics

A detailed description of statistical analysis is presented in Supplementary data, Appendix 2, available in *Age and Ageing* online.

## Results

The characteristics of participants (*n* = 845) with complete multidimensional health assessment and GPrr by baseline GS sex-specific quartiles (Q1–Q4) are shown in Supplementary data, Table S3, available in *Age and Ageing* online. Participants in Q1 were more likely to have poor or fair health, multi-morbidity, cognitive impairment and low physical activity (all *P* ≤ 0.002), compared with participants in other quartiles.

### All-cause mortality and baseline GS

At the end of 9.6-year mortality follow-up, 607 (74.7%) participants with baseline GS measurement died [261 men and 346, *P* < 0.001]. Mean survival time was 5.05 years (95% confidence interval (CI): 4.83–5.27).

Kaplan–Meier survival curves between sex-specific quartiles of baseline GS showed significant differences (*P* < 0.001) in survival (see the Supplementary data, Appendix 3, and Figure 1, panel A, available in *Age and Ageing* online). Participants in Q4 had the longest survival (mean: 6.07 years, 95% CI: 5.66–6.48) and shortest in Q1 (mean: 3.68 years, 95% CI: 3.28–4.08). In adjusted Cox models a reduced risk of all-cause mortality was observed with higher baseline GS (continuous) for all participants, men and women (Table 1). Specifically, for each kg increase in GS we observed a 3% lower risk in men, and a 4% decreased risk in women after adjustment for health, lifestyle factors and anthropometry (Model 4).

**Table 1.** Hazard ratios (HRs) for all-cause mortality by baseline GS (continuous) in the Newcastle 85+ Study

Model	Baseline GS	All participants (*n*_1_ = 813; *n*_2_ = 607)		Men (*n*_1_ = 313; *n*_2_ = 261)		Women (*n*_1_ = 500; *n*_2_ = 346)	
		HR (95% CI)	*P* value	HR (95% CI)	*P* value	HR (95% CI)	*P* value
Model 1		0.99 (0.98–1.00)	0.06	0.96 (0.94–0.98)	<0.001	0.92 (0.90–0.94)	<0.001
Model 2		0.92 (0.90–0.94)	<0.001	NA		NA	
Model 3		0.95 (0.93–0.98)	<0.001	0.97 (0.95–0.99)	0.009	0.96 (0.94–0.99)	0.007
Model 4		0.95 (0.93–0.98)	<0.001	0.97 (0.95–0.99)	0.009	0.96 (0.94–0.99)	0.007

GS, grip strength; CI, confidence intervals; Q1–Q4, quartiles of baseline GS; *n*_1_, total number of participants with baseline GS; *n*_2_, number of participants with baseline GS who died of all causes over 9.6 years. Model 1 is unadjusted. Model 2 is adjusted for sex and sex × baseline GS interaction term in all participants. Model 3 is adjusted for health-related factors (self-rated health, depressive symptoms, number of chronic diseases and cognitive status) and lifestyle (physical activity and current alcohol intake). Model 4 is additionally adjusted for anthropometry (fat mass and height). The missing values for baseline self-rated health, cognitive status, physical activity, current alcohol intake and depressive symptoms were imputed as described in Supplementary data, Appendix 1, available in *Age and Ageing* online. Model fit statistics are reported in Table S6 in Supplementary data, available in *Age and Aging* online.

### All-cause mortality and GS slopes

GS slopes (overall, negative and positive) are described in Supplementary data, Appendix 3, available in *Age and Ageing* online.

#### Overall GS slope and mortality

At the end of the 9.6-year follow-up, 421 (69.9%) participants with overall slope had died. Overall survival was 5.98 years (95% CI: 5.76–6.17). Kaplan–Meier curves between sex-specific tertiles of overall GS slope showed significant differences in survival (*P* = 0.001) (see Supplementary data, Figure 1, panel B, available in *Age and Ageing* online). Participants in the highest tertile (T3) had the shortest mean survival of 5.52 years (95% CI: 5.16–5.87), and those in T2 the longest survival (6.41 years, 95% CI: 6.05–6.76) (dashed black line). Adjusted Cox models of overall GS slope (continuous) showed a 22% increased risk of all-cause mortality with every kg/year GS change (decline) in women (*P* = 0.006), but not in men after adjustment for baseline GS, health variables, lifestyle and anthropometry (Table 2, Model 4).

**Table 2.** Hazard ratios (HRs) for all-cause mortality by overall (mean) GS slope (continuous)^a^ in the Newcastle 85+ Study

Model	All participants (*n*_1_ = 602; *n*_2_ = 421)		Men (*n*_1_ = 228; *n*_2_ = 179)		Women (*n*_1_ = 374; *n*_2_ = 242)	
	HR (95% CI)	*P* value	HR (95% CI)	*P* value	HR (95% CI)	*P* value
Model 1						
GS slope	1.10 (1.04–1.17)	0.002	1.04 (1.04–1.23)	0.4	1.16 (1.05–1.28)	0.004
Model 2						
GS slope	1.21 (1.10–1.33)	<0.001	1.06 (0.98–1.15)	0.15	1.24 (1.13–1.36)	<0.001
Baseline GS	0.95 (0.93–0.96)	<0.001	0.96 (0.94–0.98)	<0.001	0.91 (0.88–0.94)	<0.001
Model 3						
GS slope	1.19 (1.08–1.31)	<0.001	1.03 (0.94–1.13)	0.5	1.22 (1.11–1.35)	<0.001
Baseline GS	0.97 (0.95–0.99)	0.001	0.97 (0.95–0.997)	0.03	0.94 (0.92–0.98)	0.002
Model 4						
GS slope	1.19 (1.08–1.31)	<0.001	1.03 (0.94–1.13)	0.56	1.22 (1.11–1.35)	<0.001
Baseline GS	0.96 (0.94–0.98)	<0.001	0.97 (0.94–0.99)	0.01	0.95 (0.91–0.98)	0.002

GS, grip strength; CI, confidence intervals; *n*_1_, total number of participants with overall (mean) GS slope over 5 years; *n*_2_, number of participants with GS slope who died of all causes over 9.6 years.

^a^Overall (mean) slope had downward trajectory, and positive numbers represent loss (decline) in GS.

Model 1 is unadjusted. Model 2 is adjusted for sex, baseline GS (continuous) and sex × GS slope interaction term (except in men and women includes only baseline GS). Model 3 is further adjusted for health-related factors (self-rated health, depressive symptoms, number of chronic diseases and cognitive status) and lifestyle (physical activity and current alcohol intake). Model 4 is additionally adjusted for anthropometry (fat mass and height). The missing values for baseline self-rated health, cognitive status, physical activity, current alcohol intake and depressive symptoms were imputed as described in Supplementary data, Appendix 1, available in *Age and Ageing* online. Model fit statistics are reported in Table S6 in Supplementary data, available in *Age and Ageing* online.

#### Negative GS slope and mortality

Kaplan–Meier curves between sex-specific tertiles of negative GS slope showed a significant difference in survival (*P* < 0.001) (see the Supplementary data, Appendix 3, and Figure 1, panel C, available in *Age and Ageing* online). At the end of the 9.6-year follow-up, 131 (29.0%) participants with negative slopes were still alive, with overall mean survival time 6.02 years (95% CI: 5.78–6.26). Participants in T1 had the longest mean survival (6.51 years, 95% CI: 6.09–6.93), whereas those in T3 had the shortest survival (5.20 years, 95% CI: 5.78–6.26). Adjusted Cox models with negative GS slope (continuous) showed every kg/year GS decline was associated with a 16% (*P* = 0.005) and 33% (*P* = 0.01) increased risk in men and women, respectively, after adjustment for all confounders (Table 3, Model 4).

**Table 3.** Hazard ratios (HRs) for all-cause mortality by (mean) negative GS slope (continuous)^a^ in the Newcastle 85+ Study

Model	All participants (*n*_1_ = 451; *n*_2_ = 320)		Men (*n*_1_ = 183; *n*_2_ = 144)		Women (*n*_1_ = 268; *n*_2_ = 176)	
	HR (95% CI)	*P* value	HR (95% CI)	*P* value	HR (95% CI)	*P* value
Model 1						
GS slope	1.19 (1.12–1.27)	<0.001	1.13 (1.04–1.23)	0.003	1.29 (1.16–1.44)	<0.001
Model 2						
GS slope	1.31 (1.18–1.45)	<0.001	1.14 (1.05–1.23)	0.002	1.30 (1.18–1.44)	<0.001
Baseline GS	0.94 (0.92–0.96)	<0.001	0.95 (0.93–0.98)	<0.001	0.92 (0.88–0.96)	<0.001
Model 3						
GS slope	1.31 (1.17–1.46)	<0.001	1.14 (1.03–1.27)	0.01	1.32 (1.17–1.49)	<0.001
Baseline GS	0.96 (0.94–0.98)	<0.001	0.96 (0.93–0.99)	0.006	0.95 (0.91–0.99)	0.01
Model 4						
GS slope	1.31 (1.17–1.46)	<0.001	1.16 (1.05–1.29)	0.005	1.33 (1.18–1.50)	<0.001
Baseline GS	0.95 (0.93–0.98)	<0.001	0.96 (0.93–0.99)	0.006	0.94 (0.90–0.98)	0.004

GS, grip strength; CI, confidence intervals; *n*_1_, total number of participants with (mean) negative GS slope (GS decline) over 5 years; *n*_2_, number of participants with GS decline who died of all causes over 9.6 years.

^a^Negative slope (GS decline) was represented by positive numbers. Greater values represent greater loss in GS.

Model 1 is unadjusted. Model 2 is adjusted for sex, baseline GS (continuous) and sex × GS slope interaction term (except in men and women includes only baseline GS). Model 3 is additionally adjusted for health-related factors (self-rated health, depressive symptoms, number of chronic diseases and cognitive status) and lifestyle (physical activity and current alcohol intake). Model 4 is further adjusted for anthropometry (fat mass and height). The missing values for baseline self-rated health, cognitive status, physical activity, current alcohol intake and depressive symptoms were imputed as described in Supplementary data, Appendix 1, available in *Age and Ageing* online.

#### Positive GS slope and mortality

Kaplan–Meier curves between sex-specific tertiles of positive slope showed no difference in the probability of survival (*P* = 0.09) (details not shown). In fully adjusted Cox models with positive slope (continuous) (Model 4, Table S4, Supplementary data, available in *Age and Ageing* online), every kg/year increase in GS was associated with a 31% decreased risk of mortality in all participants (*P* = 0.03). We observed no significant interaction between sex and positive GS slope, thus no sex differences in survival were explored.

The results for the sensitivity analyses are shown in Supplementary data, Appendix 3 and Table S5, available in *Age and Ageing* online.

## Discussion

In this study we have shown that weaker GS at baseline and greater decline in strength over 5 years were associated with increased risk of mortality in very old men and women over the 9.6 years follow-up, independently of a range of confounding factors (multi-morbidity, cognitive impairment, physical activity and fat mass). Men's adjusted risk of mortality was increased by 16% for every kg/year of (absolute) GS lost, whereas women's risk was 33% suggesting that, although women's overall GS slopes were less steep than men's [26], every kg/year that was lost reduced women's physiological reserve and their chances for survival.

For both sexes, initial (absolute) GS was relevant for long-term survival irrespective of baseline physical activity and fat mass. The predictive value of baseline GS for long-term survival in older adults aged ≥85 has been observed previously [16, 17]. Despite differences across the studies reviewed, including length of follow-up and selection of confounders, the importance of initial GS (muscle strength) for survival over 7–10 years have been seen in the Leiden 85-plus Study (35% increased risk for those in the lowest sex-specific tertile) [16], and in the Danish 1905-Cohort study (lifespan was positively correlated with the GS intercept in both sexes) [17]. Higher GS may indicate a greater physiological reserve and resilience upon which survival in old age may be exceptionally dependent [16, 17].

In contrast, the relevance of change in GS for survival by sex (alone or in combination with initial GS) has been less clear and warrants further investigation [13, 16–23]. Steeper GS slope may reveal immediacy of death rather than age-related decline, and may be more accurate in identifying the risk of mortality in very old adults. To our knowledge, only two studies have investigated the association between GS slope and mortality in those aged ≥85 [16, 17]. Specifically, in the Leiden 85-plus Study, the highest tertile of the relative loss in GS over 4 years (from age 85 to 89) was independently associated with an increased risk of all-cause mortality over 9.5 years [16]. A faster rate of change in GS has been recognised as a predictor of mortality in Swedish twins aged 79–96 [13], in older women aged 70–79 from the Women's Health and Aging Study II irrespective of initial GS and key confounders [20], and in older Mexican American men and women [each 0.1 decrease in normalised GS (GS kg/body weight kg) was associated with a 15 and 12% increased risk of mortality, respectively] [22].

The risk of all-cause mortality over the 9.6 years follow-up in our study was unexpectedly higher in women than in men for every kg/year of (absolute) GS lost, despite men having higher baseline GS and greater annual rate of decline [26]. There are several possible explanations for this finding. We have previously reported that men experienced a linear decline in GS, whereas women's decline was non-linear—less steep slope but a slight acceleration over 5 years [26]. This acceleration may suggest a terminal decline in physical function, which may have influenced the association between GS slope and mortality that we observed in women. The Religious Order Study (participants aged 88.6 at death) reported an accelerated decline in global motor function (including GS) 2.5 years before death [29]. When we repeated analysis excluding women who died within 6 months after the last GS measurement, the results changed very little (data not shown). However, insufficient power in data may also explain the results for mortality predicted by negative GS slope in men.

A combined effect of biological, social, psychological and behavioural factors and differences between them in men and women has been proposed as possible explanation for the male–female health-survival paradox [30]. These factors may also explain the differences in type, severity and trajectories of common causes of death between men and women [30]. In the present cohort, more women than men died of cerebrovascular diseases and dementia (details not shown), suggesting a compromised overall brain health and weaker brain–muscle connections, which may have contributed to greater risk of mortality among women who were losing muscle strength. Although we controlled for baseline cognitive status in the models, cognitive trajectories and neurological changes relevant for muscle were not captured by the SMMSE. On the other hand, more men than women died of cancers—a condition that leads to an early death, leaving a sub-cohort of exceptionally robust and fit ‘survivors’ for whom the rate of change in GS (decline) for survival may be less detrimental.

Our study has several limitations: (i) we used (mean) absolute change in GS to model longitudinal data in survival analysis, and early mortality of participants with only two consecutive GS assessments (e.g. at baseline and wave 2) may have underestimated the HR; (ii) although we adjusted for multi-morbidity, severity and the rate of progression of individual diseases may have affected the probability of survival; (iii) uncontrolled confounding (e.g. type of medication and diet); (iv) older adults with initially higher GS may have been ‘preselected’ as individuals with the highest physiological reserve, and a selective loss to follow-up may have influenced our results by introducing a survival effect; (v) women were more numerous than men (500 versus 313, respectively), which may have reduced the power to establish associations in men and (vi) the results may not be generalised to other populations outside England and Wales.

The strengths of our study are: (i) we have shown that change in GS in the very old is heterogeneous—not all GS slopes were negative, and participants who gained strength had decreased risk of mortality; (ii) we started with a large cohort of very old people, and prospectively evaluated GS over 5 years in relation to long-term survival in men and women and (iii) we included a range of confounders commonly reported in the mortality literature.

To conclude, we confirmed the predictive value of baseline GS for long-term survival (~10 years) in both men and women aged 85 years, adding to importance of muscle strength for longevity in very late life. Additionally, we reported decline in GS (negative slope) as being predictive of mortality in men and women independently of initial strength and other key covariates. To confirm the biological and clinical importance of GS as a predictor of long-term survival in very old adults, the results need to be explored further in future prospective cohort studies that include a greater number of older men.

**Key points**

- We examined the association between initial level and 5-year rate of change in GS and mortality in the very old (aged ≥85).
- Every kg higher baseline GS was associated with a 3 and 4% reduced risk of 10-year mortality in men and women, respectively.
- Every kg/year GS loss was associated a 16 (men) and 33% (women) increased risk of mortality.
- Five-year increase in GS was protective of mortality.

## Supplementary Material

Supplementary Data

## References

[1] Dodds RM, Syddall HE, Cooper R et al Grip strength across the life course: normative data from twelve British studies. PLoS One 2014; 9: e113637. 25474696 10.1371/journal.pone.0113637 PMC4256164

[2] Frederiksen H, Hjelmborg J, Mortensen J, McGue M, Vaupel JW, Christensen K Age trajectories of grip strength: cross-sectional and longitudinal data among 8,342 Danes aged 46 to 102. Ann Epidemiol 2006; 16: 554–62. 16406245 10.1016/j.annepidem.2005.10.006

[3] Hicks GE, Shardell M, Alley DE et al Absolute strength and loss of strength as predictors of mobility decline in older adults: the InCHIANTI Study. J Gerontol A Biol Sci Med Sci 2012; 67A: 66–73. 10.1093/gerona/glr055 PMC3260485 21546582

[4] Rantanen T, Avlund K, Suominen H, Schroll M, Frändin K, Pertti E Muscle strength as a predictor of onset of ADL dependence in people aged 75 years. Aging Clin Exp Res 2002; 4: 10–5. 12475129

[5] Sayer AA, Kirkwood TB Grip strength and mortality: a biomarker of ageing? Lancet 2015; 386: 226–7. 25982159 10.1016/S0140-6736(14)62349-7

[6] Gale CR, Martyn CN, Cooper C, Sayer AA Grip strength, body composition, and mortality. Int J Epidemiol 2007; 36: 228–35. 17056604 10.1093/ije/dyl224

[7] Leong DP, Teo KK, Rangarajan S et al Prognostic value of grip strength: findings from the Prospective Urban Rural Epidemiology (PURE) study. Lancet 2015; 386: 266–73. 25982160 10.1016/S0140-6736(14)62000-6

[8] Cooper R, Strand BH, Hardy R, Patel KV, Kuh D Physical capability in mid-life and survival over 13 years of follow-up: British birth cohort study. Br Med J 2014; 348: g2219. 24787359 10.1136/bmj.g2219 PMC4004787

[9] Kishimoto H, Hata J, Ninomiya T et al Midlife and late-life handgrip strength and risk of cause-specific death in a general Japanese population: the Hisayama Study. J Epidemiol Community Health 2014; 68: 663–8. 24622276 10.1136/jech-2013-203611

[10] Rantanen T, Masaki K, He Q, Ross GW, Willcox BJ, White L Midlife muscle strength and human longevity up to age 100 years: a 44-year prospective study among a decedent cohort. Age (Dordr) 2012; 34: 563–70. 21541735 10.1007/s11357-011-9256-y PMC3337929

[11] Cooper R, Kuh D, Hardy R. Mortality Review Group, FALCon and HALCyon Study Teams Objectively measured physical capability levels and mortality: systematic review and meta-analysis. Br Med J 2010; 341: c4467. 20829298 10.1136/bmj.c4467 PMC2938886

[12] Visser M, Deeg DJ, Lips P, Harris TB, Bouter LM Skeletal muscle mass and muscle strength in relation to lower-extremity performance in older men and women. J Am Geriatr Soc 2000; 48: 381–6. 10798463 10.1111/j.1532-5415.2000.tb04694.x

[13] Proctor DN, Fauth EB, Hoffman L et al Longitudinal changes in physical functional performance among the oldest old: insight from a study of Swedish twins. Aging Clin Exp Res 2006; 18: 517–30. 17255642 10.1007/BF03324853

[14] Dodds RM, Granic A, Davies K, Kirkwood TB, Jagger C, Sayer AA Prevalence and incidence of sarcopenia in the very old: findings from the Newcastle 85+ Study. J Cachexia Sarcopenia Muscle 2017; 8: 229–37. 27897431 10.1002/jcsm.12157 PMC5377385

[15] Legrand D, Vaes B, Matheï C, Swine C, Degryse JM The prevalence of sarcopenia in very old individuals according to the European consensus definition: insights from the BELFRAIL study. Age Ageing 2013; 42: 727–34. 24014657 10.1093/ageing/aft128

[16] Ling CH, Taekema D, de Craen AJ, Gussekloo J, Westendorp RG, Maier AB Handgrip strength and mortality in the oldest old population: the Leiden 85-plus study. CMAJ 2010; 182: 429–35. 20142372 10.1503/cmaj.091278 PMC2842834

[17] Oksuzyan A, Maier H, McGue M, Vaupel JW, Christensen K Sex differences in the level and rate of change of physical function and grip strength in the Danish 1905-cohort study. J Aging Health 2010; 22: 589–610. 20453156 10.1177/0898264310366752 PMC2952838

[18] Metter EJ, Talbot LA, Schrager M, Conwit R Skeletal muscle strength as a predictor of all-cause mortality in healthy men. J Gerontol A Biol Sci Med Sci 2002; 57: B359–65. 12242311 10.1093/gerona/57.10.b359

[19] Buchman AS, Wilson RS, Boyle PA, Bienias JL, Bennett DA Change in motor function and risk of mortality in older persons. J Am Geriatr Soc 2007; 55: 11–9. 17233680 10.1111/j.1532-5415.2006.01032.x

[20] Xue QL, Beamer BA, Chaves PH, Guralnik JM, Fried LP Heterogeneity in rate of decline in grip, hip, and knee strength and the risk of all-cause mortality: the Women's Health and Aging Study II. J Am Geriatr Soc 2010; 58: 2076–84. 21054287 10.1111/j.1532-5415.2010.03154.x PMC3058914

[21] Hirsch CH, Buzková P, Robbins JA, Patel KV, Newman AB Predicting late-life disability and death by the rate of decline in physical performance measures. Age Ageing 2012; 41: 155–61. 22156556 10.1093/ageing/afr151 PMC3280678

[22] Peterson MD, Zhang P, Duchowny KA, Markides KS, Ottenbacher KJ, Soham AS Declines in strength and mortality risk among older Mexican American: joint modelling of survival and longitudinal data. J Gerontol A Biol Sci Med Sci 2016; 71: 1646–52. 27013398 10.1093/gerona/glw051 PMC5106852

[23] Syddall HE, Westbury LD, Dodds R, Dennison E, Cooper C, Sayer AA Mortality in the Hertfordshire Ageing Study: association with level and loss of hand grip strength in later life. Age Ageing 2016; 0: 1–6. doi:10.1093/ageing/afw222. 10.1093/ageing/afw222 PMC5500162 27932364

[24] Collerton J, Barrass K, Bond J et al The Newcastle 85+ study: biological, clinical and psychological factors associated with healthy ageing: study protocol. BMC Geriatr 2007; 7: 14. 17594470 10.1186/1471-2318-7-14 PMC1924857

[25] Collerton J, Davies K, Jagger C et al Health and disease in 85 year olds: baseline findings from the Newcastle 85+ cohort study. Br Med J 2009; 399: b4904. 10.1136/bmj.b4904 PMC2797051 20028777

[26] Granic A, Davies K, Jagger C, Kirkwood TB, Syddall HE, Sayer AA Grip strength decline and its determinants in the very old: longitudinal findings from the Newcastle 85+ Study. PLoS One 2016; 11: e0163183. 27637107 10.1371/journal.pone.0163183 PMC5026378

[27] Innerd P, Catt M, Collerton J et al A comparison of subjective and objective measures of physical activity from the Newcastle 85+ study. Age Ageing 2015; 44: 691–4. 26018999 10.1093/ageing/afv062 PMC4476851

[28] Siervo M, Prado C, Hooper L et al Serum osmolarity and haematocrit do not modify the association between the impedance index (Ht(2)/Z) and total body water in the very old: the Newcastle 85+ study. Arch Gerontol Geriatr 2015; 60: 227–32. 25288578 10.1016/j.archger.2014.09.004

[29] Wilson RS, Segawa E, Buchman AS, Boyle PA, Hizel LP, Bennett DA Terminal decline in motor function. Psychol Aging 2012; 27: 998–1007. 22612603 10.1037/a0028182 PMC3480971

[30] Case A, Paxson CH Sex differences in morbidity and mortality. Demography 2005; 42: 189–214. 15986983 10.1353/dem.2005.0011

