# Supplementary material for: Initial level and rate of change in grip strength predict all-cause mortality in very old adults
Source: Age Ageing. 2017 May 25;46(6):970–6. doi: 10.1093/ageing/afx087 (PMC5860048; doi:10.1093/ageing/afx087)
Supplement: Supplementary Data [file afx087_aa-16-1041-file002.docx]

**Supplementary Data**

**Appendix 1**

**Methods**

*Study cohort recruitment*

The sampling frame for the Newcastle 85+ Study comprised all individuals born in 1921 (aged 85 at baseline in 2006), and who were permanently registered with a participating general practice (GP) in Newcastle or North Tyneside NHS Primary Care Trusts in North-East England. General practitioners from 64 GPs were asked to exclude participants who had end stage terminal illness, and those who may pose a safety risk for a research nurse visiting alone. Based on the exclusions, 1453 people were eligible to participate in the study regardless of their health status and place of residency (residential and institution). Of those, 851 (58.6%) were recruited to multidimensional health assessment and medical records review, 188 (12.9%) to records review, and 3 (0.3%) to health assessments only [24, 25]. The analytic sample for the present study comprised 845 participants with complete data from multidimensional health assessment and medical records review who were followed for 5 years. Details about study questionnaires and assessments are available at <http://research.ncl.ac.uk/85plus/>.

*GS slopes over 5 years (absolute change) calculation*

The difference in GS from previous measurement (absolute change) was divided by the times between the measurements (approximately 1.5 years) to calculate the overall 5-year (mean) slope. Specifically, the differences in GS from baseline to wave 2 (*n* = 596); wave 2 to wave 3 (*n* = 445), and wave 3 to wave 4 (*n* = 288) were divided by the time between the waves. Positive (absolute) values indicated GS decline, and negative values indicated GS increase. The mean of the three slopes represented the overall rate of change in GS per year, which was determined for 602 (71.2%) participants (thereafter ‘overall GS slope’). Of those, 73.8% had ≥2 individual GS slopes between baseline and 5-year follow-up for the overall slope calculation. From 602 participants with the overall slope, 451 (74.9%) experienced negative slopes (thereafter ‘negative GS slope’) or constant GS decline, and 151 (25.1%) had positive slopes (GS increase) or no change in GS (thereafter ‘positive GS slope’) over 5 years.

*Description and categorisation of confounders*

Heath-related factors were self-rated health (excellent or very good/ good/ fair or poor), cognitive impairment [scoring ≤26 points on the 30-point Standardized Mini Mental Status Examination (SMMSE)], depressive symptoms [assessed by the 15-item Geriatric Depression Scale; none (0-5 points)/ mild or moderate (6-7 points)/ severe (8-15 points)] [27], and multi-morbidity (number of chronic diseases: 0-1/ 2/ ≥3). Lifestyle factors included current alcohol intake (yes/ no) and physical activity [based on self-reported intensity and frequency of physical activity per week; low (score 0-1)/ medium (score 2-6)/ high (score 7-18)] [31]. Anthropometry included height (in cm) and fat mass (in kg) calculated from demi-span and bioimpendance formula, respectively) [32].

*Imputed confounders*

Of 813 participants with baseline GS, most had <3% missing data for selected confounders except for physical activity (3.9%) and fat mass (10.8%). The following missing values were imputed with the reference (ref) category: self-rated health (*n* = 9, ref: poor/fair), cognitive status at baseline (*n* =1; ref: impaired), physical activity (*n* = 29, ref: high), current alcohol intake (*n* = 4, ref: yes), depressive symptoms (*n* = 57, ref: severe). Fat mass (*n* = 88) and height (*n* =1) (continuous) were imputed with sex-specific means.

We used multiple linear regression to assess the confounders for multicollinearity and by inspecting VIF tolerance, eigenvalues and the condition index.

**Appendix 2**

**Statistics**

*Descriptive statistics*

Participants characteristics by sex-specific quartiles of baseline GS were compared using ANOVA for normally distributed continuous variables, Kruskal-Wallis for ordered and non-normally distributed continuous variables, and χ^2^ tests for categorical variables. All statistics were reported at two-tailed α ≤ 0.05.

*Survival analysis*

*Unadjusted probability of survival by quartiles of GS at baseline and tertiles of GS slopes over 5 years*

The Kaplan-Meier test was used to assess relative risk of all-cause mortality in sex-specific quartile of GS at baseline (*n* = 813), and by sex-specific tertiles of GS slopes. Only significant differences in the probability of survival for overall (*n* = 602) and negative slope tertiles (*n* = 451) were reported and evaluated further with Cox proportional hazard models [the positive slope tertiles (*n* = 151) were not significant]. We also examined whether the mean survival time increased proportionally across the GS quartiles/tertiles. The time interval was calculated as the period between the date of baseline GS measurement (2006/2007) and the date of death or being censored on 6 January 2016. The Kaplan-Meier plots were inspected for proportionality of risk in relation to baseline GS quartiles and tertiles of GS slopes over 9.6 years of mortality follow-up.

*Adjusted probability of survival by baseline GS and GS slopes (continuous)*

Cox proportional hazards models were then used to estimate hazard ratios (HRs), with 95% confidence intervals (CI), for the risk of all-cause mortality associated with baseline GS and GS slopes (continuous); the proportional hazards assumption was assessed via log-minus-log and partial residual plots. Four models were explored; (Model 1) unadjusted; (Model 2) adjusted for sex and interaction terms where appropriate (sex*baseline GS for baseline GS models, and sex*GS slope for slope models); (Model 3) further adjusted for baseline health and lifestyle factors (see above), and (Model 4) further adjusted for anthropometry. Models were stratified by sex if significant sex*slope interactions were observed. To assess the model fit, we used the log-likelihood statistics and χ^2^ distribution to test the difference in log-likelihoods between the models. The improvements of the models with baseline GS and GS slopes (continuous) by the log-likelihood statistics are summarized in Table S6. Pseudo R^2^ estimates were based on Cox & Smell’s algorithm.

All analyses were performed using IBM SPSS Statistics software version 21 (IBM Corporation, Armonk, NY, USA).

*Sensitivity analysis*

Cox proportional hazard models were fitted with the sex-specific tertiles of the overall and negative slopes. Relative risk of mortality did not differ across tertiles of positive slopes, therefore Cox proportional hazard models were not explored. For overall and negative slope the highest tertiles (T3) representing highest loss per year were used as a referent.

We contrasted the relative risks of all-cause mortality obtained from Cox proportional hazard models in which covariates were not imputed with results with the imputed covariates.

**Appendix 3**

**Results**

*GS slopes over 5 years (absolute change)*

The overall GS slope had downward trajectory, a mean change of 0.86 kg (SD = 1.63) per year [1.1 (1.94) kg/year in 228 men and 0.71 (1.39) kg/year in 374 women] over 5 years. The negative slope (GS decline) was seen in 451 participants, a mean change of 1.44 (1.35) kg/year [1.70 (1.51) kg/year in 183 men, and 1.26 (1.19) kg/year in 268 women]. The positive slope (GS increase) was observed in 151 participants, a mean change of -0.86 (1.07) kg/year [-1.35 (1.50) kg/year in 45 men, and -0.67 (0.74) kg/year in 106 women]. Overall GS slopes were steeper in men than in women, especially in the negative GS slope group.

*Unadjusted probability of survival by quartiles of GS at baseline and tertiles of GS slopes over 5 years*

The Kaplan-Meier survival curves between sex-specific quartiles of GS showed significant difference in survival (Mantel-Cox χ2 (3) = 70.3, *P* < 0.001), which increased proportionally across the quartiles (mean survival time in Q1: 3.68 years, 95% CI 3.28-4.08; Q2: 4.94 years, 95% CI 4.51-5.38; Q3: 5.56 years, 95% CI 5.13-5.99, and Q4: 6.07 years, 95% CI 5.66-6.48). The differences in survival between Q1 and Q4 of GS were apparent within the first two years of mortality follow-up, and continued thereafter (see the Supplementary data, Figure 1, panel A). Similarly, we observed significant differences in survival across tertiles of overall (Mantel-Cox χ2 (2) = 13.15, *P* = 0.001) and negative GS slope (Mantel-Cox χ2 (2) = 22.9, *P* < 0.001). Specifically, the probability of cumulative survival (mean years) increased proportionally from T3 (high) to T1 (low) of negative GS slope (T3: 5.20, 95% CI 4.79-5.61; T2: 6.36, 95% CI 5.97-6.75, and T1: 6.51, 95% CI 6.07-6.93) (see Supplementary data, Figure 1, panel C).

**Results for sensitivity analysis**

*All-cause mortality associated with sex-specific baseline GS quartiles and tertiles of GS slopes*

The results observed with baseline GS as a continuous variable were corroborated in the Cox proportional hazard models with the GS categorized in sex-specific quartiles. Participants in the lowest GS quartile (Q1) had a 55% increased risk of mortality (95% CI 1.21-1.99, *P* = 0.001) after adjustment for all covariates compared with the highest GS quartile (Q4) (Model 4; Supplementary Table 3). Those in the lowest sex-specific tertile of overall GS slope (T1) had a 30% reduced risk of mortality (95% CI 0.55-0.90, *P* = 0.006) compared with participants in T3 in the fully adjusted model (Model 4). Participants in the lowest sex-specific tertile of negative slope (T1) had a 45% decreased risk of mortality (95% CI 0.41-0.73, *P* < 0.001) compared with those experiencing a steep GS decline (T3).

*All-cause mortality associated with GS (baseline and 5-years slopes) with non-imputed covariates*

The HRs remained mostly unchanged when models were fitted excluding imputed values for covariates. For example, in the fully adjusted model (Model 4) every kg/year decline in negative GS slope (continuous) was associated with a 29% increased risk of mortality in all participants (95% CI 1.13-1.46, *P* < 0.001) compared with 31% increased risk observed in the imputed model (details not shown).

**Supplementary Tables**

**Table S1.** Sex-specific quartiles of GS at baseline in the Newcastle 85+ Study

___________________________________________________________________________

Quartiles (kg) Men Women

___________________________________________________________________________

Q1 (low) ≤19.63 ≤10.25

Q2 19.64-24.37 10.26-13.50

Q3 24.38-29.23 13.51-16.30

Q4 (high) ≥29.24 ≥16.31

___________________________________________________________________________

GS, grip strength; Q1 to Q4, quartiles

**Table S2.** Sex-specific tertiles of (mean) GS slopes in the Newcastle 85+ Study

____________________________________________________________________________________________________________________

Overall GS slope^a^ Negative GS slope^b^ Positive GS slope^c^ ______________________________________________________________________________________________________________________________

Tertiles (kg) Men Women Tertiles (kg/year) Men Women Tertiles (kg/year) Men Women

______________________________________________________________________________________________________________________________

T1 ≤0.48 ≤0.18 T1 ≤0.89 ≤0.64 T3 -0.52-0 -0.20-0

T2 0.49-1.57 0.19-1.03 T2 0.90-1.83 0.65-1.28 T2 -1.59- -0.53 -0.61- -0.21

T3 ≥1.58 ≥1.04 T3 ≥1.84 ≥1.29 T1 ≤-1.59 ≤-0.62

______________________________________________________________________________________________________________________________

GS, grip strength; T1 to T3, tertiles.

^a^Overall GS slope had downward trajectory and was represented by positive numbers. T3 was used as a referent.

^b^Negative GS slope (decline) was represented by positive numbers. T3 was used as a referent.

^c^Positive GS slope (increase or no change) was represented by negative numbers. Tertiles were reverse-coded, and T1 (high increase) was used as a referent.

**Table S3.** Characteristics of participants (*n* = 845) by baseline sex-specific quartiles of GS

____________________________________________________________________________________________________________________

Characteristic Q1 Q2 Q3 Q4 *P* value

____________________________________________________________________________________________________________________

*n* 208 202 201 202

Sex % (*n*)

women 62.0 (129) 60.9 (123) 61.7 (124) 61.4 (124)

*Health-related factors*

Self-rated health % (*n*) <0.001

excellent/very good 19.8 (64) 23.2 (75) 26.0 (84) 31.0 (100)

good 22.3 (68) 25.2 (77) 26.9 (82) 25.6 (78)

poor/fair 39.8 (70) 27.8 (49) 18.8 (33) 13.6 (24)

Cognitive status <0.001

impaired (0-25 SMMSE) 42.2 (94) 26.0 (58) 19.2 (43) 12.6 (28)

normal (26-30) 19.2 (113) 24.4 (144) 26.8 (158) 29.5 (174)

Depressive symptoms (GDS) 0.002

no depression (0-5 score) 21.2 (127) 24.9 (149) 24.9 (149) 29.0 (174)

mild/moderate (6-7) 32.6 (31) 27.4 (26) 28.4 (27) 11.6 (11)

severe (8-16) 30.6 (19) 21.0 (13) 32.3 (20) 16.1 (10)

Multi-morbidity 0.002

0-1 diseases 21.6 (51) 23.3 (55) 26.7 (63) 28.4 (67)

2 diseases 20.0 (49) 24.1 (59) 26.9 (66) 29.0 (71)

3 and more 32.5 (108) 26.5 (88) 21.7 (72) 19.3 (64)

*Lifestyle factors*

Physical activity <0.001

low 52.0 (89) 24.6 (42) 13.5 (23) 9.9 (17)

moderate 24.4 (83) 24.1 (82) 28.2 (96) 23.2 (79)

high 10.3 (28) 24.9 (68) 28.6 (78) 36.3 (99)

Alcohol intake 0.002

yes 22.7 (109) 22.9 (110) 29.3 (141) 25.2 (121)

no 30.1 (94) 27.2 (85) 18.3 (57) 24.4 (76)

*Anthropometry*

height (cm), M (SD) 159.06 (8.08) 160.54 (7.51) 161.84 (7.19) 163.42 (7.87) <0.001

fat mass (kg), M (SD) 17.62 (7.69) 16.79 (7.74) 19.67 (7.31) 21.19 (7.59) <0.001

____________________________________________________________________________________________________________________

GDS, Geriatric Depression Scale; SMMSE, Standardized Mini-Mental State Examination

^a^ANOVA (with Tukey’s honestly significant difference post-hoc test) and t-tests for normally distributed continuous variables, Kruskal-Wallis for ordered and non-normally distributed continuous variables, and χ^2^ tests for categorical variables.

**Table S4.** Hazard ratios (HRs) for all-cause mortality by (mean) positive GS slope (continuous)^a^ in the Newcastle 85+ Study

___________________________________________________________________________

All participants

Model (*n*_1_ = 151; *n*_2_ = 101) _____________________________________________________________________

HR (95% CI) *P* value

___________________________________________________________________________

Model 1

*GS slope* 0.73 (0.62-0.86) <0.001

Model 2

*GS slope* 0.70 (0.51-0.97) 0.03

*Baseline GS*  0.98 (0.94-1.03) 0.43

Model 3

*GS slope* 0.70 (0.50-0.99) 0.04

*Baseline GS* 1.03 (0.98-1.08) 0.24

Model 4

*GS Slope* 0.69 (0.49-0.97) 0.03

*Baseline GS*  1.03 (0.68-1.57) 0.24

___________________________________________________________________________

GS, grip strength, CI, confidence intervals.

^a^Positive slope (GS increase) was represented by negative numbers. Greater (absolute) numbers represent increase in GS.

Model 1 is unadjusted. Model 2 includes sex, baseline GS (continuous) and sex*GS slope interaction term. Model 3 is additionally adjusted for health-related factors (self-rated health, depressive symptoms, number of chronic diseases, and cognitive status) and lifestyle (physical activity and current alcohol intake). Model 4 is further adjusted for anthropometry (fat mass and height). No significant interaction between sex*slope was found, thus the models stratified by sex were not employed.

The missing values for baseline self-rated health, cognitive status, physical activity, current alcohol intake, and depressive symptoms were imputed with the reference (ref) category, and height and fat mass were imputed with sex-specific means as described in Appendix 1.

**Table S5.** Hazard ratios (HRs) for all-cause mortality by baseline GS (sex-specific quartiles), overall, and negative slope (sex-specific tertiles) in the Newcastle 85+ Study

____________________________________________________________________________________________________________________

Categorized GS Model 1 Model 2 Model 3 Model 4

______________________________________________________________________________________________________________________________

Sex-specific quartiles of GS (baseline)^a^, *n*_1_ = 813

HR (95% CI) *P* value HR (95% CI) *P* value HR (95% CI) *P* value HR (95% CI) *P* value

____________________________________________________________________________________________________

Q1 (low), *n*_2_ = 185 2.22 (1.77-2.78) <0.001 not applicable 1.50 (1.17-1.91) 0.001 1.55 (1.21-1.99) 0.001

Q2, *n*_2_ = 158 1.46 (1.16-1.84) 0.001 1.21 (0.96-1.54) 0.11 1.24 (0.97-1.58) 0.08

Q3, *n*_2_ = 134 1.13 (0.89-1.44) 0.32 1.07 (0.83-1.36) 0.06 1.07 (0.84-1.37) 0.6

Q4 (high), *n*_2_ = 130 1 (reference) 1 (reference) 1 (reference)

____________________________________________________________________________________________________

Sex-specific tertiles of overall GS slope^b^, *n*_1_ = 602

HR (95% CI) *P* value HR (95% CI) *P* value HR (95% CI) *P* value HR (95% CI) *P* value

____________________________________________________________________________________________________

T1 (low), *n*_2_ = 134 0.77 (0.61-0.97) 0.03 0.75 (0.59-0.95) 0.02 0.79 (0.62-1.01) 0.06 0.70 (0.55-0.90) 0.006

T2 , *n*_2_ = 129 0.68 (0.54-0.86) 0.001 0.68 (0.54-0.86) 0.001 0.75 (0.59-0.95) 0.02 0.69 (0.55-0.88) 0.003

T3 (high), *n*_2_ = 158 1 (reference) 1 (reference) 1 (reference) 1 (reference)

____________________________________________________________________________________________________

Sex-specific tertiles of negative GS slope^c^, *n*_1_ = 451

HR (95% CI) *P* value HR (95% CI) *P* value HR (95% CI) *P* value HR (95% CI) *P* value

__________________________________________________________________________________________________

T1 (low), n_2_ = 93 0.57 (0.43-0.74) <0.001 0.56 (0.43-0.74) <0.001 0.61 (0.46-0.81) 0.001 0.55 (0.41-0.73) <0.001

T2, n_2_ = 103 0.66 (0.51-0.85) 0.002 0.65 (0.50-0.84) 0.001 0.71 (0.54-0.93) 0.001 0.66 (0.50-0.86) 0.003

T3 (high), n_2_ = 124 1 (reference) 1 (reference) 1 (reference) 1 (reference)

_____________________________________________________________________________________________________________________________

GS, grip strength, CI, confidence intervals; Q1 to Q4, quartiles of baseline GS; T1 to T3 tertiles of GS slope. *n*_1_, total number of participants with baseline GS or GS slope; *n*_2_, number of participants with baseline GS or GS slope who died of all causes over 9.6 years.

^a^For (sex-specific) GS quartiles (at baseline) cut-offs see TableS1.

^b^For (sex-specific) tertiles of overall GS slope cut-offs see Table S2 (left panel). Overall GS slope had downward trajectory represented by positive numbers.

^c^For (sex-specific) tertiles of negative GS slope cut-offs see Table S2 (middle panel). GS decline (negative slope) was represented by positive numbers.

Model 1 is unadjusted. Model 2 is adjusted for baseline GS. Model 3 is additionally adjusted for health-related factors (self-rated health, depressive symptoms, number of chronic diseases, and cognitive status) and lifestyle (physical activity and current alcohol intake). Model 4 is further adjusted for anthropometry (fat mass and height).

The missing values for baseline self-rated health, cognitive status, physical activity, current alcohol intake, and depressive symptoms were imputed as described in Appendix 1.

**Table S6**. Log-likelihoods statistics for the goodness of fit for Cox regression models with baseline GS and GS slopes (continuous)

______________________________________________________________________________________________________________________________

-2LL (original) -2LL (model) χ2 (df) (overall) *P* χ2 (df) (change) *P* pseudo R^2^

______________________________________________________________________________________________________________________________

Cox regression models with baseline GS (Table 1)

*All participants*

Model 1 7569.615 7565.868 3.675 (1) 0.055 3.747 (1) 0.053 0.002

Model 2 7569.615 7480.708 90.074 (3) <0.001 88.907 (3) <0.001 0.083

Model 3 7569.615 7384.765 208.882 (13) <0.001 184.850 (13) <0.001 0.170

Model 4 7569.615 7372.248 220.326 (15) <0.001 197.367 (15) <0.001 0.182

*Men*

Model 1 2710.666 2688.850 22.020 (1) <0.001 21.816 (1) <0.001 0.059

Model 2 N/A

Model 3 2710.66 2663.875 52.648 (11) <0.001 46.791 (11) <0.001 0.099

Model 4 2710.666 2652.032 62.545 (13) <0.001 58.634 (13) <0.001 0.117

*Women*

Model 1 4012.124 3967.954 44.261 (1) <0.001 44.170 (1) <0.001 0.058

Model 2 N/A

Model 3 4012.124 3885.582 146.37 (11) <0.001 126.54 (11) <0.001 0.195

Model 4 4012.124 3882.575 149.487 (13) <0.001 129.549 (13) <0.001 0.207

______________________________________________________________________________________________________________________________

Cox regression models with GS slope (overall) (Table 2)

*All participants*

Model 1 5042.903 5034.295 9.363 (1) 0.002 8.609 (1) 0.003 0.012

Model 2 5014.576 4956.805 63.479 (4) <0.001 57.771 (4) <0.001 0.087

Model 3 5014.576 4908.419 119.402 (14) <0.001 106.157 (14) <0.001 0.157

Model 4 5014.576 4901.734 125.811 (16) <0.001 112.842 (16) <0.001 0.171

*Women*

Model 1 2688.362 2680.754 8.184 (1) 0.004 7.607 (1) 0.006 0.011

Model 2 2662.207 2624.858 40.621 (2) <0.001 37.349 (12) <0.001 0.068

Model 3 2662.207 2571.072 105.838 (12) <0.001 91.135 (12) <0.001 0.203

Model 4 2662.207 2569.387 108.073 (14) <0.001 92.820 (14) <0.001 0.219

______________________________________________________________________________________________________________________________

GS, grip strength; -2LL, -2 log-likelihood; df, degrees of freedom. -2LL (original) represents the -2LL of the null model; -2LL (model) represents the -2LL of the fitted model, and -2LL (change) represents the change in -2LL from the previous model. Pseudo R^2^ is based on Cox & Smell’s algorithm. Cox regression models with GS slope (overall) in men were not significant.

**Figure**


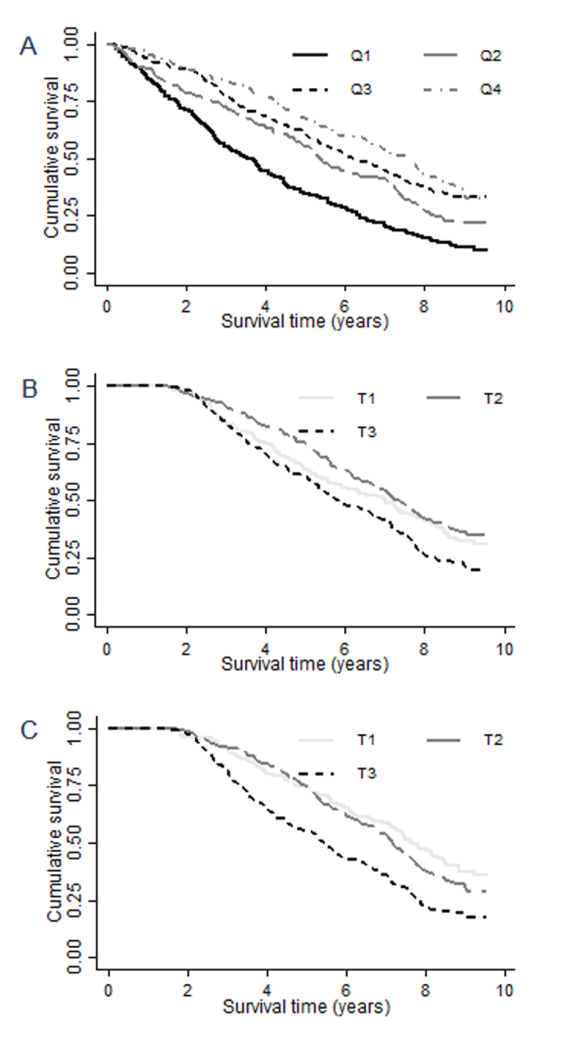


**Figure 1.** Kaplan-Meier plot for probability of survival by sex-specific quartiles of baseline GS (A), sex-specific tertiles of overall GS slope (B), and sex-specific tertiles of negative GS slope (C).

**Figure legend**

**Figure 1.** Kaplan–Meier plot of the probability of survival by level and rate of decline in grip strength (GS). We observed statistically significant differences in survival across sex-specific quartiles of baseline GS over 9.6 years of follow-up in the Newcastle 85+ Study. Men and women in the highest quartile of baseline GS had a higher probability of survival (Q4, dashed grey line; panel A) from all causes of death compared with participants in other quartiles. Participants in the highest (sex-specific) tertile of overall GS slope (T3, dashed black line; panel B) had the shortest survival compared with other tertiles. Also, participants in the lowest tertile of negative GS slope (T1, solid light grey line; panel C) had the highest probability of survival compared with others.
